# Fatty Acid Composition and Contents of Fish of Genus *Salvelinus* from Natural Ecosystems and Aquaculture

**DOI:** 10.3390/biom12010144

**Published:** 2022-01-16

**Authors:** Michail I. Gladyshev, Alexander A. Makhrov, Ilia V. Baydarov, Stanislava S. Safonova, Viktor M. Golod, Sergey S. Alekseyev, Larisa A. Glushchenko, Anastasia E. Rudchenko, Vladimir A. Karpov, Nadezhda N. Sushchik

**Affiliations:** 1 Institute of Biophysics, Krasnoyarsk Scientific Center, Siberian Branch, Russian Academy of Sciences, 660036 Krasnoyarsk, Russia; labehe@ibp.ru; 2 Institute of Biophysics SB RAS, Siberian Federal University, 660041 Krasnoyarsk, Russia; loraglushchenko@gmail.com (L.A.G.); rudchenko.a.e@gmail.com (A.E.R.); vlkarpov2@gmail.com (V.A.K.); 3 A. N. Severtsov Institute of Ecology and Evolution, Russian Academy of Sciences, 119071 Moscow, Russia; makhrov12@mail.ru; 4 Department of Animal Science, Russian State Agrarian University—Moscow Timiryazev Agricultural Academy, 127550 Moscow, Russia; paajarviru@gmail.com (I.V.B.); sfalij@yandex.ru (S.S.S.); 5 Federal Selection and Genetic Center of Fish Farming, 188514 Ropsha, Russia; ropshatrout@yandex.ru; 6 Koltzov Institute of Developmental Biology, Russian Academy of Sciences, 119334 Moscow, Russia; alekseyev@mail.ru

**Keywords:** eicosapentaenoic acid, docosahexaenoic acid, Arctic charr, brook trout, nutritive value

## Abstract

Fatty acids (FA) of muscle tissue of *Salvelinus* species and its forms, *S. alpinus*, *S. boganidae*, *S. drjagini*, and *S. fontinalis*, from six Russian lakes and two aquacultures, were analyzed. Considerable variations in FA compositions and contents were found, including contents of eicosapentaenoic and docosahexaenoic acids (EPA and DHA), which are important indicators of fish nutritive value for humans. As found, contents of EPA+DHA (mg·g^−1^ wet weight) in muscle tissue of *Salvelinus* species and forms varied more than tenfold. These differences were supposed to be primarily determined by phylogenetic factors, rather than ecological factors, including food. Two species, *S. boganidae* and *S. drjagini*, had the highest EPA+DHA contents in their biomass and thereby could be recommended as promising species for aquaculture to obtain production with especially high nutritive value. Basing on revealed differences in FA composition of wild and farmed fish, levels of 15-17-BFA (branched fatty acids), 18:2NMI (non-methylene interrupted), 20:2NMI, 20:4n-3, and 22:4n-3 fatty acids were recommended for verifying trade label information of fish products on shelves, as the biomarkers to differentiate wild and farmed charr.

## 1. Introduction

Fish are evidently a very important natural bioresource for humans. However, wild catches reach their upper limit of 100 10^6^ t y^−1^ [1,2], and in the recent decade, fish supply for human nutrition from aquaculture became equal to that of wild catch [3,4]. Fish is known to be the source of many valuable nutrients: proteins, lipids, microelements, etc. However, the uniqueness of fish as a food source is determined by the contents of long-chain polyunsaturated fatty acids of the omega-3 family (LC-PUFA), namely of eicosapentaenoic acid (20:5n-3, EPA) and docosahexaenoic acid (22:6n-3, DHA). Indeed, a contribution of fish to the global consumption of protein is ~6% [5], while the contribution to global EPA+DHA consumption is > 97% [6]. LC-PUFA are known to be essential components of human diet, which provide numerous health benefits, including prevention of cardiovascular diseases and neural disorders [7,8,9,10]. The World Health Organization, as well as many national health organizations, recommended personal daily consumption 0.5–1.0 g of EPA+DHA [7,11,12,13,14,15]. It is well known that the main food source of EPA and DHA for human diet is fish [5,6,15,16,17,18,19,20]. Nevertheless, it is also known that contents of EPA+DHA vary ca. 400-fold in different fish species, and thereby some fish with the LC-PUFA contents below 1 mg g^−1^ of wet weight (WW) are not the food source, which really provides the recommended daily dose of PUFA of 0.5–1 g [18,21,22,23]. Thus, continual improvement of databases on EPA and DHA contents in diverse food fish species from different locations and habitats is critical in provision of accurate recommendations on the healthy LC-PUFA intake for individuals and public health officials [11,24,25]. 

As is known, there are two main groups of factors, which determine LC-PUFA contents in fish biomass: phylogenetic and ecological factors [23,26,27]. In general, phylogenetic factors are believed to overweigh the ecological. For instance, the maximum value of EPA+DHA contents in muscle tissue of species of the order Cypriniformes is 4.71 mg g^−1^ WW [28], while in species of the order Salmoniformes, it is 32.78 mg·g^−1^, and in spite a great intra-taxa variability, no one of cyprinids has the maximum value as high as salmonid [23,29]. This difference is evidently caused by phylogenetic differences in lipid storage patterns: cyprinids have their lipid storage in intraperitoneal adipose tissue, while salmonids can store the lipids also in muscle tissue [30]. Nevertheless, although Salmoniformes have inherently higher LC-PUFA than many other fish taxa, in certain unfavorable ecological conditions, such as low food, water pollution, etc., they also can have EPA+DHA contents far below their genetically determined potential maximum value [31,32]. For instance, wild *Coregonus lavaretus* from different habitats had intra-species variations of EPA+DHA contents from 1.87 to 16.61 mg g^−1^ [32]. These differences in the nutritive value of conspecific fish should be taken into account for commercial fisheries as well for aquaculture. In aquaculture, food and other rearing conditions are evidently the principal determinants of LC-PUFA level in edible biomass of reared species. However, for aquaculture species, a selection of strains capable of accumulating high quantities of EPA and DHA even under their comparatively low contents in feed, e.g., in a case of substitution of fish oils by vegetable oils, appeared to be very important task [33,34,35,36,37,38].

Fish of genus *Salvelinus* are known to be valuable targets of commercial fisheries and aquaculture. Among salmonids, *Salvelinus* species have especially high morphological and ecological diversity [39,40,41], which is probably caused by unstable environmental conditions in their habitats—Arctic lakes [42]. We hypothesize, that within such diverse taxa, there is a considerable diversity in EPA and DHA contents in biomass. Thus, the aim of our study was to evaluate fatty acid (FA) composition and contents of species and forms of genus *Salvelinus* in different Russian lakes to expand database on nutritive value of wild fish. Moreover, we aimed to compare FA composition and contents of wild fish of this genus with those reared in aquaculture. This comparison was intended to reveal the following: (1) new species and forms for aquaculture with a high potential to accumulate EPA and DHA in their biomass; (2) marker fatty acids for differentiating wild and aquaculture fish on shelves to check trade labels information for consumers.

## 2. Materials and Methods 

### 2.1. Studied Lakes

**Figure 1:**
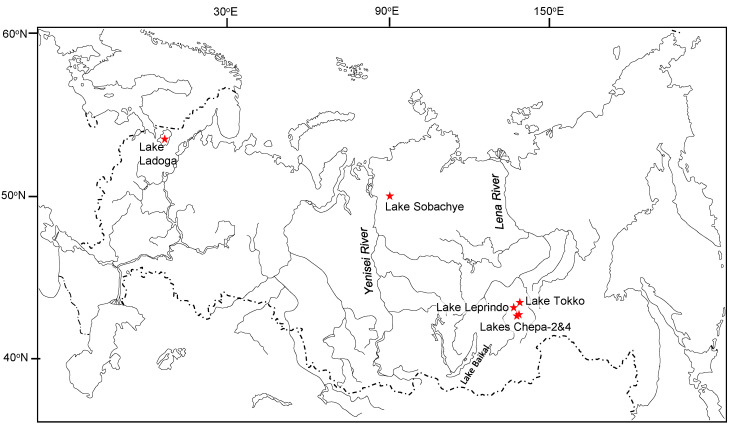
Map of sample sites (stars).

All sampled water bodies (Table 1) were oligotrophic (except nearly mesotrophic Lake Ladoga) and in summer–autumn had low surface water temperature, 5.5–15.0 ℃. A map of the sampled water bodies is given in Figure 1. Locations of sample sites were chosen from different biomes, where populations of charrs with different body sizes were previously studied.

### 2.2. Fish Sampling

Fish were caught, euthanized, and sampled in accordance with Federal rules and the BioEthics Protocol on Animal Care, approved by the Siberian Federal University (approval code: No. 33215-2014). Species of genus *Salvelinus*, collected in diverse water bodies and in aquaculture, and sample sizes are given in Table 2. Although feeding habits of these species were well known from the literature [22,45,46,47], the stomach contents of some specimens were taken for microscopic analyses to check their food items (Table 2), and for fatty acid (FA) analysis (see below).

Boganid charr *Salvelinus boganidae* Berg, 1926, were caught in Lake Sobachye. Boganid charr is benthivore–piscivore [22] (Table 2). 

Dryagin’s charr *Salvelinus drjagini* Logaschev, 1940, were caught in Lake Sobachye. Dryagin’s charr is piscivore [47] (Table 2).

Diverse forms of Arctic charr *Salvelinus alpinus* L., including large forms from lakes Ladoga, Chepa-2, and Chepa-4, small forms from lakes Sobachye (goggle-eyed charr) and Tokko, and dwarf forms from lakes Tokko and Bol’shoe Leprindo (B. Leprindo), were caught in 6 Russian lakes (Table 2), and also were obtained from aquaculture of Federal Selection and Genetic Center of Fish Farming in the Ropsha Village (Ropsha farm). In the Ropsha farm, *S. alpinus* from Lake Ladoga was reared. Arctic charr forms were piscivore, piscivore–benthivore, insectivore–piscivore and planktivore [22,45,46,48] (Table 2). Small and dwarf forms from Lake Tokko were pooled for the following FA analysis. It should be noted that *S. boganidae* and *S. drjagini* are close to or conspecific with *S. alpinus*.

Brook trout *Salvelinus fontinalis* (Mitchill, 1814) were obtained from an aquaculture of Russian State Agrarian University—Moscow Timiryazev Agricultural Academy. 

### 2.3. Fatty Acid Analysis

Fatty acid analyses are described elsewhere [49]. Briefly, lipids were extracted simultaneously with mechanical homogenization with chloroform/methanol mixture (2:1, *v*/*v*) 3 times. The dried lipids were hydrolyzed under reflux at 90 °C for 10 min in methanolic sodium hydroxide solution with concentration of 8 mg/mL. Then, the mixture was added with an excess methanolic solution of 3% sulfuric acid and refluxed at 90 °C for 10 min to produce fatty acid methyl esters (FAMEs). The mixture was twice washed with portions of NaCl saturated solution, and FAMEs were extracted with a portion of hexane. FAMEs were analyzed with a gas chromatograph equipped with a mass spectrometer detector (model 6890/5975C, Agilent Technologies, Santa Clara, CA, USA) and a 30 m long, 0.25 mm internal diameter capillary HP-FFAP column. Detailed description of the instrumental conditions has been given earlier, see [50]. Data were collected and analyzed using Chemstation Software (Agilent Technologies, USA). Peaks of FAMEs were identified by their mass spectra, comparing them to those in the integrated database NIST 2005 and to those in the standard 37-FAMEs mixture (U-47885, Supelco, Bellefonte, PA, USA). We used areas of the identified FAMEs to calculate two data types, proportional percentages of the total (%), and contents per unit of wet weight (mg·g^−^^1^). For data of the first type, areas of all identified FAMEs, excluding that of 19:0 (used as an internal standard), were summed and each area was divided to the sum of FAMEs. For data of the second type, FAMEs were quantified according to a peak area of the internal standard, 19:0-FAME (Sigma-Aldrich, St. Louis, MO, USA), which was added as a chloroformic solution to samples prior to the lipid extraction, after addition of the first portion of chloroform/methanol mixture. For the content calculation, we used a known mass of the internal standard added to a sample, an area of the 19:0-FAME peak, and wet weight of a muscle tissue sample. We address hereafter the data of the first type as percentages or levels, and data of the second type as contents only. 

### 2.4. Statistics

One-way ANOVA with Tukey HSD post hoc test, Kruskal–Wallis test, and multivariate canonical correspondence analysis (CCA) [51] were calculated conventionally, using STATISTICA software, version 9.0 (StatSoft, Inc., Tulsa, OK, USA). Only normally distributed variables (Kolmogorov–Smirnov one-sample test for normality) were included in ANOVA, while other variables were compared using the non-parametric Kruskal–Wallis test.

## 3. Results

**Figure 2:**
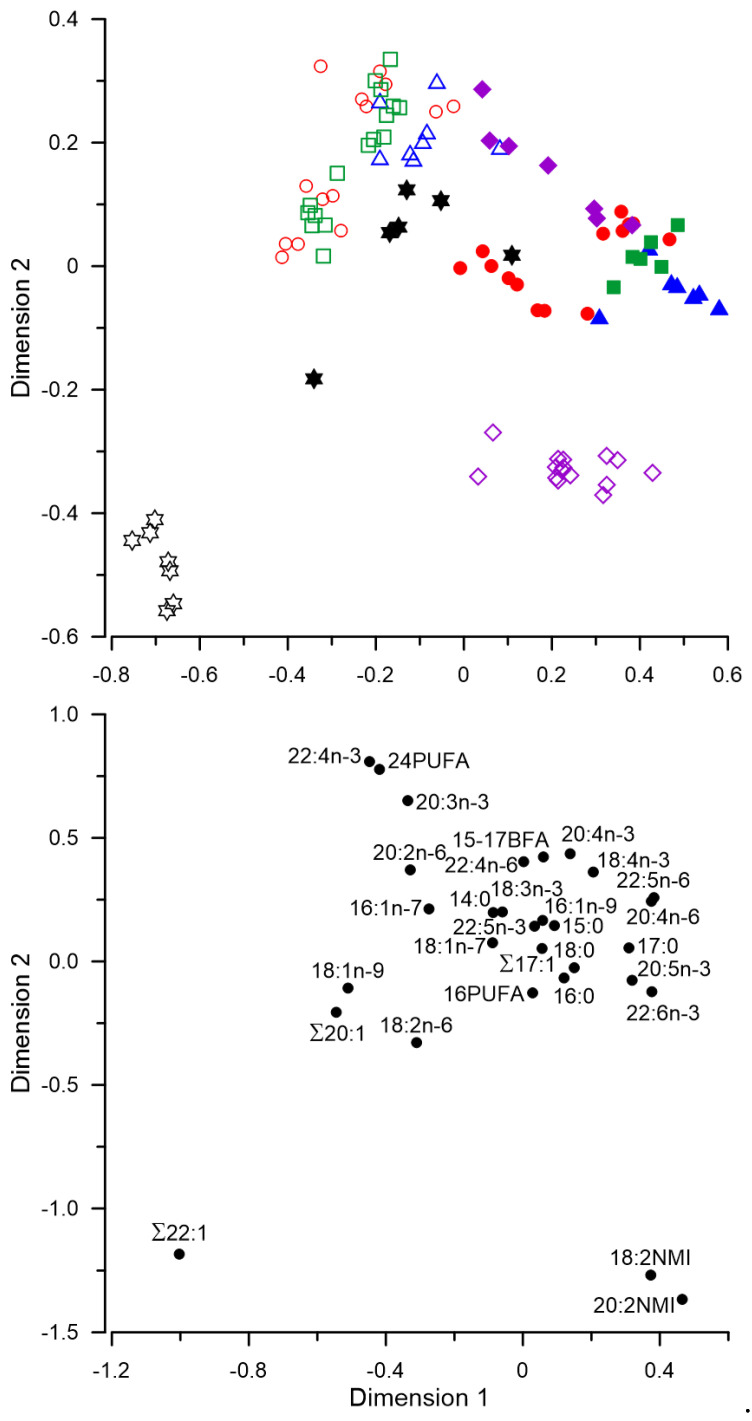
Canonical correspondence analysis of levels of fatty acids (% of the total) in species of genus *Salvelinus*: open circles—boganid charr *S. boganidae*; open squares—Dryagin’s char *Salvelinus drjagini*; open triangles—goggle-eyed charr (*Salvelinus alpinus* complex; closed circles—Arctic charr *Salvelinus alpinus* from Lake Tokko; closed triangles—*S. alpinus* from Lake Leprindo; closed squares—*S. alpinus* from Lake Chepa-2; closed diamonds—*S. alpinus* from Lake Chepa-4; closed stars—*S. alpinus* from Lake Ladog; open stars—*S. alpinus* from the Ropsha farm; open diamonds—brook trout *Salvelinus fontinalis* from the aquaculture of Timiryazev Academy. Dimension 1 and Dimension 2 represented 43.3% and 20.8% of inertia, respectively.

The canonical correspondence analysis (CCA) of FA percentages in muscle tissue of fish revealed a considerable partitioning of some groups (Figure 2). Along Dimension 1, which represented the largest proportion of inertia, most overall differences in FA composition were found between farmed *S. alpinus* and wild *S. alpinus* from Lake Leprindo and Lake Chepa-2 (Figure 2). The differences along Dimension 1 were primarily due to the contrast between levels of ∑22:1 and many other acids, such as 20:2NMI, 22:6n-3, 22:5n-6, etc. (Figure 2). Along Dimension 2, with the twice lower part of inertia, the most differences were between farmed *S. alpinus* and wild fish from Lake Sobachye (Figure 2). These differences along Dimension 2 were primarily due to the contrast between levels of 20:2NMI, 18:2NMI, and ∑22:1, on the one hand, and many other acids, such as 22:4n-3, 24PUFA, and 20:3n-3, etc., on the other hand (Figure 2).

The overall differences in FA composition of fish, revealed by CCA, were specified for each FA by ANOVA or Kruskal–Wallis tests. *S. drjagini* from Lake Sobachye tended to have the highest mean level of 22:4n-3 (Table 3). The goggle-eyed form of *S*. *alpinus* had the highest mean level of 20:2n-6 and tended to have the highest mean levels of 14:0 and 24PUFA, but tended to have the lowest mean level of ∑17:1 (Table 3). *S. alpinus* from Lake Tokko tended to have the highest mean level of 20:4n-3 (Table 3). *S. alpinus* from Lake Leprindo tended to have the highest mean levels of 18:0, 22:5n-6, and 22:6n-3 (Table 3). *S. alpinus* from Lake Chepa-2 tended to have the lowest mean level of 14:0 (Table 3). *S. alpinus* from Lake Chepa-4 had the highest mean level of 15-17BFA and tended to have the highest mean levels of 16:1n-9, ∑17:1, 18:3n-3, and 22:4n-6, but tended to have the lowest mean level of ∑22:1 (Table 3). *S. alpinus* from Lake Ladoga tended to have the highest mean level of 18:1n-7 (Table 3). Farmed *S. alpinus* had the highest mean levels of 18:1n-9 and ∑20:1, and tended to have the highest mean level of 18:2n-6, but tended to have the lowest mean levels of 16:0, 18:0, 20:4n-6, and 22:5n-3 (Table 3). *S. fontinalis* had the highest mean level of 16:0 and tended to have the highest mean levels of 18:2NMI, 20:2NMI, and 20:5n-3, but had the lowest level of 18:3n-3 and tended to have the lowest mean levels of 18:1n-7, 18:4n-3, 20:3n-3, 22:4n-6, and 24PUFA (Table 3). The aquaculture fish, *S. alpinus* and *S. fontinalis*, had significantly lower mean levels of 15-17BFA and 20:4n-3 and tended to have the lower mean level of 22:4n-3 compared with those of the wild fish (Table 3). 

**Figure 3:**
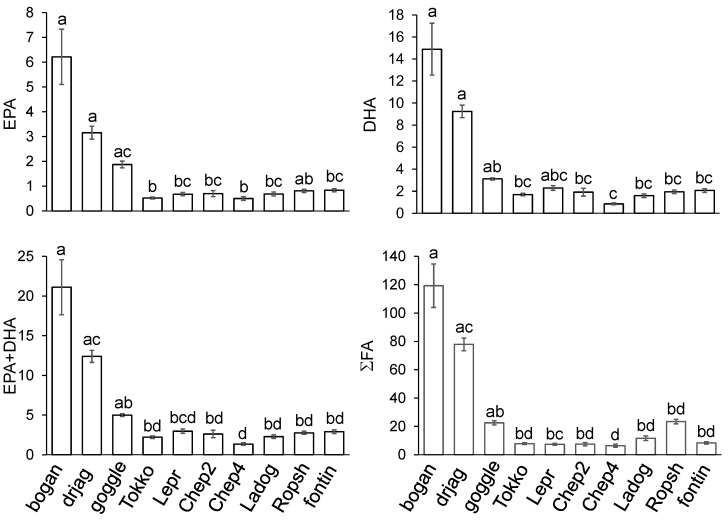
Mean values of contents (mg·g^−1^ WW) of eicosapentaenoic (EPA) and docosahexaenoic (DHA) fatty acids and their sum (EPA+DHA), and sum of total fatty acids (∑FA) in muscle tissue of studied fish: boganid charr *Salvelinus boganidae* (bogan. number of samples, *n* = 14), Dryagin’s charr *Salvelinus drjagini* (drjag, *n* = 16), goggle-eyed charr (*Salvelinus alpinus* complex) (goggle, *n* = 8), all from Lake Sobachye, Arctic charr *Salvelinus alpinus* from Lake Tokko (Tokko, *n* = 14), Lake Leprindo (Lepr, *n* = 7), Lake Chepa-2 (Chep2, *n* = 6), Lake Chepa-4 (Chep4, *n* = 7), Lake Ladoga (Ladog, *n* = 6) and Ropsha farm (Ropsh, n = 7), and brook trout *Salvelinus fontinalis* from the aquaculture of Timiryazev Academy (fontin, *n* = 15). Means labelled with the same letter are not significantly different at *p* < 0.05 according to Kruskal–Wallis test. Bars represent standard errors.

*S. boganidae* and *S. drjagini* from Lake Sobachye had significantly higher mean contents of EPA than *S. alpinus* from other lakes and *S. fontinalis* (Figure 3). *S. boganidae* and *S. drjagini* from Lake Sobachye also had significantly higher mean contents of DHA than *S. alpinus* from other lakes (except Lake Leprindo) and the farm, and *S. fontinalis* (Figure 3). Sum contents of EPA+DHA and total FA of *S. boganidae* and *S. drjagini* from Lake Sobachye were significantly higher than those of charr from other lakes (except non-significant difference between *S. drjagini* and *S. alpinus* from Lake Leprindo) and from the farm and the aquaculture (Figure 3). 

**Figure 4:**
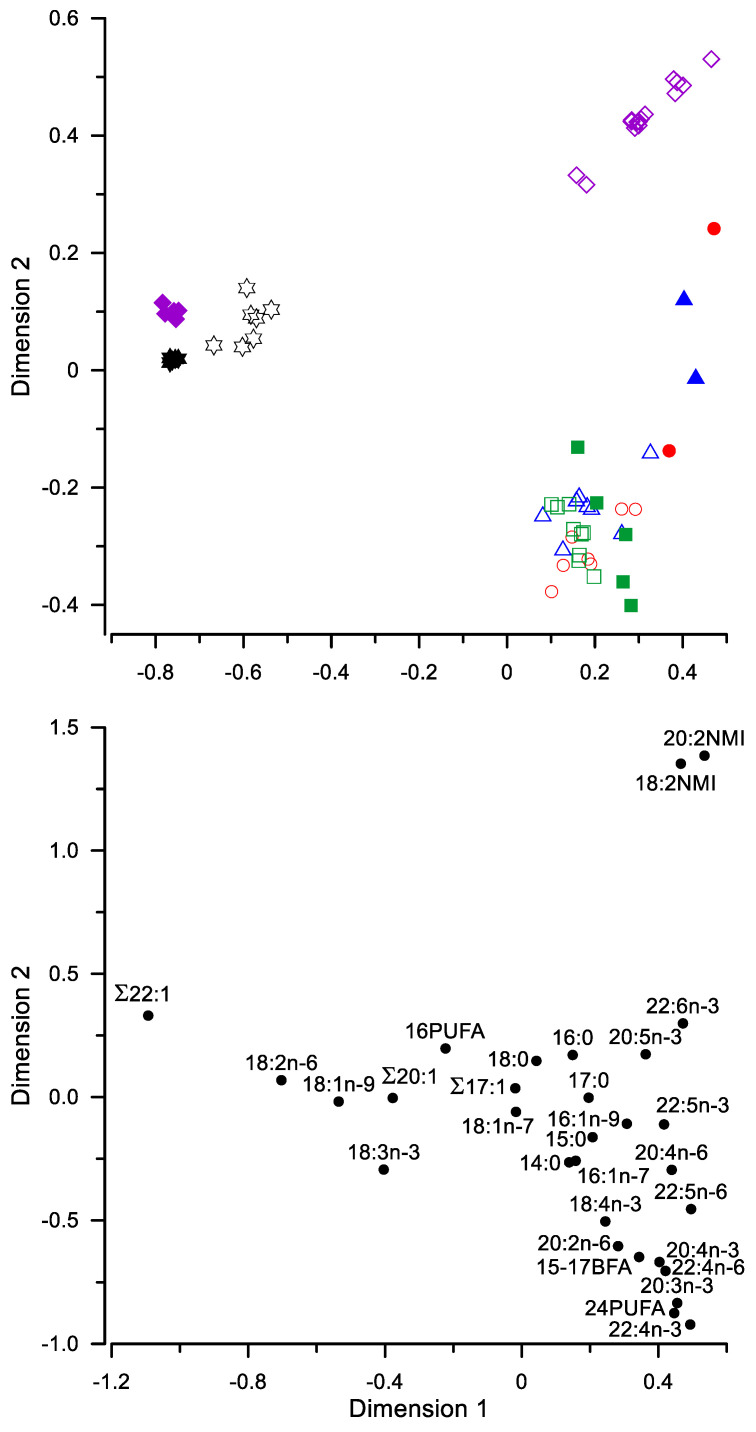
Canonical correspondence analysis of levels of fatty acids (% of the total) in fish species of genus *Salvelinus* (open symbols) and their food (closed symbols): circles—boganid charr *S. boganidae*; triangles—goggle-eyed charr (*S. alpinus* complex); squares—Dryagin’s char *S. drjagini*; stars—Arctic charr *S. alpinus* from the Ropsha farm; diamonds—brook trout *S. fontinalis* from the aquaculture of Timiryazev Academy. Dimension 1 and Dimension 2 represented 58.4% and 26.8% of inertia, respectively.

The canonical correspondence analysis of FA percentages in the tissue of some fish and their food demonstrated a considerable partitioning of some of them (Figure 4). Along Dimension 1, which represented the largest proportion of inertia, the most overall differences in FA composition were found between *S. fontinalis* and their diet (Figure 4). In contrast, there were no such considerable differences along Dimension 1 between wild fish *S. boganidae*, *S. drjagini*, and the goggle-eyed form of *S*. *alpinus* and their gut contents, as well as between the farmed *S*. *alpinus* and their diet (Figure 4). Moreover, there were no considerable differences along Dimension 1 between diets of the farmed *S*. *alpinus* and *S. fontinalis*, reared in aquaculture (Figure 4). The differences along Dimension 1 between the two formulated diets and gut contents of wild fish, as well as between muscles of wild fish and *S. fontinalis* on the one hand, and the farmed *S*. *alpinus*, on the other hand were primarily due to the contrast between levels of ∑22:1 and many other acids, such as 20:2NMI, 18:2NMI, and 22:6n-3, etc. (Figure 4). Along Dimension 2, with the twice lower part of inertia, most differences were between wild fish and *S. fontinalis* from the aquaculture (Figure 4). These differences along Dimension 2 were primarily due to the contrast between levels of 20:2NMI and 18:2NMI vs. many other acids, such as, 22:4n-3, 24PUFA, and 20:3n-3, etc. (Figure 4).

**Figure 5:**
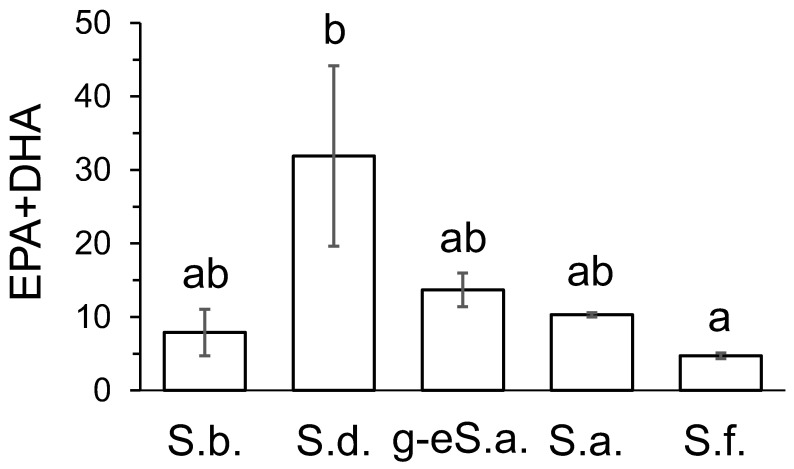
Mean values of contents (mg·g^−1^dry weight) of sum of eicosapentaenoic and docosahexaenoic fatty acids (EPA+DHA), in food of studied fish: gut content of boganid charr *Salvelinus boganidae* (S.b. number of samples, *n* = 2); Dryagin’s char *Salvelinus drjagini* (S.d., *n* = 5); goggle-eyed charr (the form of *Salvelinus alpinus* complex) (g-eS.a., *n* = 2); all from Lake Sobachye; diet of Arctic charr *Salvelinus alpinus* from the Ropsha farm (S.a. *n* = 5); diet of brook trout *Salvelinus fontinalis* from the aquaculture of Timiryazev Academy (S.f., *n* = 5). Means labelled with the same letter are not significantly different at *p* < 0.05 according to Kruskal–Wallis test. Bars represent standard errors.

The overall differences in FA composition of fish food, revealed by CCA, were specified for each FA by ANOVA or Kruskal–Wallis tests. Gut contents of *S. drjagini* had significantly higher mean level of 14:0 and tended to have the highest mean levels of 16:0, 18:4n-3, 20:2n-6, 20:4n-3, and 24PUFA, than those in food of the other fish (Table 4). Gut contents of the goggle-eyed form of *S*. *alpinus* tended to have the highest mean level of 18:1n-7. Diet of the farmed *S. alpinus* had significantly higher mean levels of 18:2n-6 and 18:3n-3 than that of the other fish and tended to have the highest mean level of 16PUFA (Table 4). Diet of *S. fontinalis* tended to have the highest mean levels of ∑20:1 and ∑22:1, but had the lowest mean level of 16:0, and tended to have the lowest mean level of 20:4n-6 (Table 4). Food (gut contents) of wild fish, *S. boganidae*, *S. drjagini*, and the goggle-eyed form of *S*. *alpinus* had significantly higher mean levels of 17:0, ∑17:1, 20:5n-3, 22:5n-6, and 22:5n-3, and tended to have higher mean levels of 15:0, 16:1n-9, 16:1n-7, 15-17BFA, 18:4n-3, 20:2n-6, 20:4n-6, 20:3n-3, 20:4n-3, 22:4n-3, and 24PUFA, compared with those of formulated diets of aquaculture fish, *S. alpinus* and *S. fontinalis* (Table 4). In turn, diets of the aquaculture fish had significantly higher mean level of 18:2n-6 and tended to have higher mean levels of 18:1n-9, 18:3n-3, ∑20:1, and ∑22:1, compared with those of the food of the wild fish (Table 4). Total content of fatty acids, mg per g of dry weight, tended to be the highest in diet of *S. alpinus* and the lowest in gut contents of *S. boganidae* (Table 4). Sum EPA+DHA content tended to be the highest in food of *S. drjagini* (Figure 5).

## 4. Discussion

The studied fish of genus *Salvelinus* varied significantly regarding contents of EPA+DHA in muscle tissue, which is the main indicator of their nutritive value for humans. Range of the variations of EPA+DHA contents was from 20.1 ± 3.5 mg·g^−1^ WW in *S. boganidae* to 1.4 ± 0.2 mg·g^−1^ WW in *S. alpinus* from Lake Chepa-4. Data from the literature on EPA and DHA content in *Salvelinus* species from other water bodies [25,52,53,54] fall in this range. Regarding the threshold of EPA+DHA content in edible fish biomass of 1 mg·g^−1^ WW [18,22], fish of genus *Salvelinus* are the valuable product for human nutrition, but their nutritive value was found to vary ~14-fold. A disclosing of mechanisms, responsible for such variations, is believed to be useful for providing of consumers with the valuable food fish through commercial fishery and aquaculture. 

Farmed *S. alpinus* in our study had mean EPA+DHA contents nearly similar to those of wild *S. alpinus* from Lake Ladoga, i.e., from the original habitat of the farmed fish. Goggle-eyed form of *S. alpinus* from Lake Sobachye tended to have higher LC-PUFA contents, than the farmed *S. alpinus*, and contents of EPA and DHA in food of the goggle-eyed form tended to be higher than that of the farmed fish. In the literature, there are data on significantly higher EPA and DHA contents in farmed *S. alpinus* compared with wild fish, in spite of higher contents of these LC-PUFA in potential food in one of wild habitats [52].

Differences in EPA and DHA in fish, as well as in other fatty acids, are known to be caused by ecological and genetic factors (e.g., [23,27]). All studied lakes were oligotrophic and unpolluted, except the nearly mesotrophic Lake Ladoga, which is subjected to an anthropogenic pollution [44]. Indeed, mesotrophic conditions and anthropogenic pollution could decrease LC-PUFA contents in fish biomass (e.g., [31,55]). However, in the present study *S. alpinus* from Lake Ladoga had EPA and DHA contents similar to those of fish from oligotrophic and unpolluted lakes. Thus, no explicit evidence on the effect of lake trophy or anthropogenic pollution on LC-PUFA contents in *Salvelinus* was found, probably because of the limited number of studied lakes.

Besides, among ecological factors, food is known to be an important determinant of fatty acid composition of consumers (“you are what you eat”). Indeed, in the present study of *Salvelinus* species, FA composition of gut contents of wild fish were, in general, related with those of their muscle tissue, as well as FA composition of food of the farmed *S*. *alpinus*. However, FA composition of *S. fontinalis* in aquaculture did not show such close relation with that of their food. Diet of *S. fontinalis* tended to have the highest mean levels of ∑20:1 and ∑22:1—markers of marine copepods [56,57]—and tended to have the lowest mean level of 20:4n-6, which is the marker of organic matter of terrestrial origin [58] and had comparatively low level in marine fish [41]. The above peculiarities of FA composition are common characteristics of formulated aquaculture feed, which is primarily prepared from marine pelagic fish, such as Peruvian anchovy [37]. The same FA markers were also characteristic of formulated feed of the farmed *S*. *alpinus*. However, the FA profile of the farmed *S*. *alpinus* was comparatively close to that of their food, while the FA profile of *S. fontinalis* from aquaculture differed dramatically from that of its formulated feed. For instance, in spite of nearly similar FA composition and contents of diets, farmed *S. alpinus* and *S. fontinalis* had significantly different levels of 18:1n-9, ∑20:1, and 20:5n-3. These differences might be determined genetically, because *S. fontinalis* is known as the most diverged species of genus *Salvelinus* [59].

Nevertheless, differences between FA composition of fish and their food, contradicting to the statement “you are what you eat”, especially regarding LC-PUFA, seem to be a common phenomenon [22,60,61], confirmed in our study. Besides the above conspicuous difference between FA profiles of *S. fontinalis* and their food, it was found that the highest EPA+DHA contents was characteristic of gut contents of *S. drjagini*, while the highest contents of these LC-PUFA in muscle tissue was characteristic of *S. boganidae*. 

The increase in LC-PUFA contents in fish biomass compared with that in their food [22,60,61] could take place due to an ability to selective accumulation of EPA and DHA or to their synthesis from the precursor, ALA, which are genetically determined features [35,62,63,64,65,66]. Genetic (phylogenetic) factors were found to be more strong determinants for FA profiles of aquatic animals, including fish than environmental factors [23,26,67,68]. Since the ability of fish to accumulate EPA+DHA in their muscle tissue, i.e., in edible biomass is strongly controlled by genetic factors, there is a potential opportunity to select species or strains with the high EPA and DHA contents for rearing in aquaculture for obtaining products with the increased nutritive value [33,34,36,37,65]. According to data, obtained in the present study, *S. boganidae* and *S. drjagini* had significantly higher potential to accumulate EPA and DHA in their biomass than *S. alpinus* and *S. fontinalis*. Thus, an introduction of these two species, *S. boganidae* and *S. drjagini*, in aquaculture seems to be very desirable for obtaining of production with especially high nutritive value. 

*S. boganidae* and *S. drjagini* had higher contents of EPA and DHA than all studied populations of *S. alpinus,* including the goggle-eyed form inhabiting Lake Sobachye together with these two species. These differences of LC-PUFA contents in the species from Lake Sobachye might be determined by their origination from two different phylogenetic lines of genus *Salvelinus* [69,70]. The ability of *S. boganidae* and *S. drjagini* to accumulate the high contents of EPA and DHA in their muscle tissue, likely is an adaptive feature, fixed in their genotypes, allowing them to occupy their specific ecological niches. Firstly, it may be the adaptation to hunting in the large deep oligotrophic lake, which implies fast continuous swimming. Indeed, an importance of DHA for fast continuous swimming of certain fish taxa was recently found using a meta-analysis [23]. An importance of DHA for successful hunting was also demonstrated [71]. In aquaculture, *S. alpinus* fed with vegetable oils with low EPA and DHA had a significantly lower swimming speed, compared with fish fed with fish oil with a high LC-PUFA contents [72]. Physiological and biochemical mechanisms underlying the relation of DHA content and swimming rate consist in the following. DHA chains in phospholipids of cell membranes are extremely flexible and have very low potential barriers for rotation, providing extremely high molecular lateral pressure [71,73]. The high lateral pressure supports higher activity of membrane-associated enzymes, such as Na^+^, K^+^-ATPase, providing higher action potential in excitable cells and fibers of muscle tissue [73,74,75]. 

Besides the importance for fast continuous swimming, the higher contents of LC-PUFA in muscle tissue of certain fish taxa may play another physiological role. Species of the order Salmoniformes, accumulate their storage lipids not only in intraperitoneal adipose tissue, but in muscle tissue (e.g., [30]). These lipids from muscles, including EPA and DHA, accumulated before a reproductive season, are transferred into gonads (e.g., [76]). Embryos, which develop at lower water temperature and have longer period of development, require greater specific amounts LC-PUFA to form cell membranes of their relatively small cells, and their parents need to store higher amounts of EPA and DHA in muscle tissue [77]. Lake Sobachye is situated considerably farther to the north than the other studied water bodies, and embryogenesis of *Salvelinus* forms and species in this lake seems to be longer, demanding the higher contents of EPA and DHA. 

In any case, if the higher contents of EPA and DHA in muscle tissue of *S. boganidae* and *S. drjagini* are caused by their style of feeding or/and by spawning, the following question arises: will this ability to accumulate LC-PUFA remain in aquaculture? Or, in other words, as follows: is the genetic determination of high EPA and DHA contents in muscles strong enough to continue after changing of wild life style (swimming, hunting, spawning) to cage life? Evidently, this question can be answered only after an introduction of these two species, *S. boganidae* and *S. drjagini*, in aquaculture. However, in the case of the introduction, the above ecological factors, supporting the high contents of LC-PUFA, should be taken into account. The opportunity for *Salvelinus* species to have in aquaculture as high content of EPA and DHA as in wild conditions is supported by our finding that *S. alpinus* in Lake Ladoga, farmed in Ropsha farm, had similar values of these LC-PUFA in the wild habitat and in the aquaculture. 

In last decades, choice of fish products by consumers is increasingly influenced by the origin of the fish, and mislabeling of fish products, e.g., wild-caught products instead of farm-raised products, by unscrupulous retailers to increase profits becomes a considerable problem [78,79]. FA profiles can be useful indicators for verifying label information of fish products [49,78,79]. In the present study, farmed *S. alpinus* and *S. fontinalis* had, in their biomass, 18:2NMI and 20:2NMI, which were absent in muscle tissue of wild fish. Besides, both fish from the farm and the aquaculture had significantly lower levels of bacterial fatty acids, 15-17-BFA (0.2–0.3% vs. 1.1–2.5% in wild fish), and 20:4n-3 (0.4% vs. 1.3–2.9% in wild fish), and 22:4n-3 (0.0% vs. 0.1–1.1% in wild fish). Two latter fatty acids may be concerned as intermediate compounds indicative for conversion of C18 to C20-22 PUFA [47,80]. As known, increased EPA and DHA content in the formulated feed down regulated expression of key genes for the PUFA conversion in farmed Atlantic salmon [81]. Increased endogenous levels of EPA and DHA in salmon hepatocytes also reduced desaturation rates of C18 to C20-22 PUFA along with reducing expression of the Δ6 desaturase gene [82]. However, in the current study contents of EPA and DHA sum in formulated feed did not exceed than that in the natural diet. In addition, contents of EPA and DHA in muscle tissues of the studied fish from aquaculture did not exceed those in the wild charr. On the other hand, the aquaculture fish evidently had balanced and plentiful feed to provide their constant growth, i.e., regular and constant supply of the PUFA with diet. In contrast, food supply of the fish, especially piscivore forms, from natural oligotrophic habitats, is variable and sometimes might be scarce. We assume that the occasional food shortage leads to better ability of the wild charr to retain dietary EPA and DHA and/or to convert the shorter acids to long-chain PUFA. Thus, 20:4n-3 and 22:4n-3, as well as 18:2NMI, 20:2NMI, and 15-17-BFA, could be used as the biomarkers for differentiating farmed and wild charr.

## 5. Conclusions

Contents of EPA+DHA in muscle tissue of *Salvelinus* species and forms varied considerably, from 1.4 ± 0.2 to 20.1 ± 3.5 mg·g^−1^ WW. These differences were supposed to be primarily determined by phylogenetic, rather than ecological factors. Two species, *S. boganidae* and *S. drjagini*, had the highest EPA+DHA contents in their biomass and thereby could be recommended as promising species for aquaculture to obtain production with especially high nutritive value. For verifying trade label information of fish products on shelves, levels of 15-17-BFA, 18:2NMI, 20:2NMI, 20:4n-3, and 22:4n-3 fatty acids could be used as the biomarkers to differentiate farmed and wild charr. 

## Figures and Tables

**Table 1:** Description of studied water bodies: locations of sample sites; surface area (*A*, km^2^); average depth (*h*_av_, m); maximum depth (*h*_max_, m); water temperature at 0–5 m (*t*, ℃) in periods of sampling (summer–autumn); pH; and references (Ref.).

Water Body	Location	*A*	*h*_av_	*h*_max_	*t*	pH	Ref.
Lake Sobachye	69°01′ N, 91°05′ E	99	nd	162	6.5	nd	[32,43]
Lake Ladoga	60°50′ N, 31°33′ E	17,800	47	230	9.5	7.4	[44]
Lake Tokko	57°11′ N, 119°41′ E	0.63	nd	40	11.0	8.0	[45]
Lake B. Leprindo	56°37′ N, 117°31′ E	17	nd	67	15.0	8.1	[46]
Lake Chepa-2	56°59′ N, 119°69′ E	0.07	nd	10	6.2	6.5	*
Lake Chepa-4	56°59′ N, 119°66′ E	0.06	nd	12	5.5	6.7	*

nd—no data; *—our unpublished data.

**Table 2:** The basic biological and sampling information on *Salvelinus* species from Russian water bodies and aquaculture, 2016-2020: *n*—number of sampled individuals; Month—month of catching; L—total length, cm (mean ± SE); W—total weight, g (mean ± SE); Food—items found in stomachs; Am—Amphipoda; T—Trichoptera larvae; C.s—*Coregonus sardinella*; C.t.—*Coregonus tugun*; P.p.—*Phoxinus phoxinus*; Z—zooplankton; Ch.—Chironomidae (pupae, larvae); P.per.—*Phoxinus percnurus*; C.p.—*Cottus* cf. *poecilopus;* CF—commercial feed (pellets) (arranged in cells in the descending order of their mass fraction in gut content); nd—no data.

Species Name	Common Name	Water Body	*n*	Month	L	W	Food
*S. boganidae*	Boganid charr	Lake Sobachye	14	Sep.	53.7 ± 2.9	2198 ± 369	Am, T,
*S. drjagini*	Dryagin’s charr	Lake Sobachye	16	Jul.–Sep.	64.2 ± 0.0	3528 ± 493	C.s.
*S. alpinus*	Goggle-eyed charr	Lake Sobachye	8	Sep.	26.0 ± 0.0	177 ± 23	C.t., C.s.
*S. alpinus*	Arctic charr, small form	Lake Tokko	7	Aug.	26.6 ± 0.5	214 ± 14	P.p.
*S. alpinus*	Arctic charr, dwarf form	Lake Tokko	7	Aug.	16.7 ± 0.4	42 ± 4	Z, Ch.
*S. alpinus*	Arctic charr	Lake B. Leprindo	7	Aug.	15.1 ± 0.2	28 ± 2	Z
*S. alpinus*	Arctic charr	Lake Chepa-2	6	Sep.	38.5 ± 0.8	607 ± 42	Z, P.per.
*S. alpinus*	Arctic charr	Lake Chepa-4	7	Sep.	43.0 ± 0.9	619 ± 44	P.per, Z, C.p
*S. alpinus*	Arctic charr	Lake Ladoga	6	Oct.	87.7 ± 3.4	3171 ± 520	nd
*S. alpinus*	Arctic charr	Ropsha farm	7	Sep.	70.4 ± 4.3	1311 ± 916	CF
*S. fontinalis*	Brook trout	Aquaculture	15	Jun.	15.9 ± 5.5	41 ± 4	CF

**Table 3:** Mean values of percentages of fatty acids (% of the total ± standard error) in muscle tissue of studied fish: boganid charr *Salvelinus boganidae* (number of samples, *n* = 14), Dryagin’s charr *Salvelinus drjagini* (*n* = 16), goggle-eyed charr (*Salvelinus alpinus* complex) (*n* = 8), all from Lake Sobachye, Arctic charr *Salvelinus alpinus* from Lake Tokko (*n* = 14), Lake Leprindo (*n* = 7), Lake Chepa-2 (*n* = 6), Lake Chepa-4 (*n* = 7), Lake Ladoga (*n* = 6) and Ropsha farm (*n* = 7), brook trout *Salvelinus fontinalis* from the aquaculture of Timiryazev Academy (*n* = 15). Normally distributed variables are compared by ANOVA and Tukey HSD post hoc test; the other variables are marked with asterisk * and are compared by Kruskal–Wallis test. Means labelled with the same letter are not significantly different at *p* < 0.05 according to the relevant test.

	*S. boganidae* Lake Sobachye	*S. drjagini* Lake Sobachye	Dwarf *S. alpinus* Lake Sobachye	*S. alpinus* Lake Tokko	*S. alpinus* Lake Leprindo	*S. alpinus* Lake Chepa-2	*S. alpinus* Lake Chepa-4	*S. alpinus* Lake Ladoga	*S. alpinus* Ropsha Farm	*S. fontinalis* Aquaculture
14:0	2.8 ± 0.1^A^	3.5 ± 0.2^AB^	3.9 ± 0.2^B^	2.8 ± 0.3^A^	2.1 ± 0.3^AC^	1.5 ± 0.2^C^	2.0 ± 0.2^AC^	2.4 ± 0.6^AC^	1.9 ± 0.1^AC^	2.2 ± 0.2^AC^
15:0 *	0.4 ± 0.1^ABCD^	0.3 ± 0.0^ACD^	0.3 ± 0.0^ABD^	0.4 ± 0.0^AB^	0.3 ± 0.0^ABD^	0.4 ± 0.0^ABD^	0.4 ± 0.0^B^	0.3 ± 0.0^ABCD^	0.2 ± 0.0^CD^	0.3 ± 0.0^D^
16:0	15.1 ± 0.6^A^	16.3 ± 0.4^A^	16.2 ± 0.3^AB^	19.5 ± 0.6^B^	17.7 ± 0.6^AB^	17.4 ± 0.5^AB^	14.7 ± 1.4^ABC^	16.3 ± 1.0^A^	10.9 ± 0.7^C^	23.6 ± 1.0^D^
16:1n-9 *	0.4 ± 0.0^ABCD^	0.4 ± 0.0^ABD^	0.5 ± 0.0^AC^	0.6 ± 0.0^AC^	0.3 ± 0.0^BD^	0.4 ± 0.0^ABD^	1.1 ± 0.2^C^	0.4 ± 0.0^ABCD^	0.3 ± 0.0^D^	0.4 ± 0.0^AD^
16:1n-7 *	8.0 ± 0.3^A^	7.9 ± 0.3^A^	4.4 ± 0.7^AB^	4.3 ± 0.5^BD^	2.4 ± 0.2^BC^	3.0 ± 0.2^BC^	5.5 ± 0.5^ACD^	8.2 ± 1.4^AD^	3.8 ± 0.5^BD^	3.6 ± 0.2^BD^
15-17BFA	1.3 ± 0.0^A^	1.5 ± 0.1^A^	1.4 ± 0.0^A^	1.5 ± 0.1^A^	1.3 ± 0.1^A^	1.4 ± 0.1^A^	2.5 ± 0.4^B^	1.1 ± 0.1^A^	0.3 ± 0.0^C^	0.2 ± 0.0^C^
16PUFA *	0.2 ± 0.0^AB^	0.2 ± 0.0^AB^	0.4 ± 0.1^ABC^	0.1 ± 0.0^B^	0.1 ± 0.0^BD^	0.4 ± 0.0^ACD^	1.1 ± 0.2^C^	0.7 ± 0.2^AC^	0.4 ± 0.0^AC^	0.7 ± 0.1^C^
17:0	0.2 ± 0.0^AC^	0.2 ± 0.0^AB^	0.3 ± 0.0^D^	0.4 ± 0.0^E^	0.4 ± 0.0^EF^	0.4 ± 0.0^EF^	0.4 ± 0.0^EF^	0.3 ± 0.0^DF^	0.1 ± 0.0^C^	0.2 ± 0.0^BD^
∑17:1	0.1 ± 0.0^A^	0.2 ± 0.0^BD^	0.0 ± 0.0^C^	0.3 ± 0.0^BE^	0.1 ± 0.0^AD^	0.3 ± 0.0^BE^	0.4 ± 0.0^E^	0.3 ± 0.0^BE^	0.1 ± 0.0^AD^	0.1 ± 0.0^AC^
18:0	3.6 ± 0.3^A^	3.6 ± 0.1^A^	2.5 ± 0.2^B^	5.1 ± 0.3^CD^	5.4 ± 0.4^C^	3.9 ± 0.3^A^	3.8 ± 0.3^A^	4.0 ± 0.2^AD^	2.2 ± 0.1^B^	4.0 ± 0.1^A^
18:1n-9	22.8 ± 1.3^A^	21.6 ± 1.1^AB^	17.1 ± 0.9^BF^	10.4 ± 1.1^CE^	5.7 ± 0.7^C^	6.7 ± 0.5^C^	9.0 ± 0.7^CE^	20.6 ± 2.0^AB^	37.2 ± 1.0^D^	12.4 ± 0.5^EF^
18:1n-7	3.6 ± 0.1^ACD^	3.4 ± 0.1^ABC^	2.9 ± 0.1^AB^	3.3 ± 0.4^AB^	2.4 ± 0.3^B^	3.0 ± 0.2^AB^	4.4 ± 0.3^CD^	4.5 ± 0.2^D^	2.8 ± 0.1^AB^	2.6 ± 0.1^B^
18:2NMI *	0.0 ± 0.0^A^	0.0 ± 0.0^A^	0.0 ± 0.0^A^	0.0 ± 0.0^A^	0.0 ± 0.0^A^	0.0 ± 0.0^AB^	0.0 ± 0.0^A^	0.0 ± 0.0^A^	0.1 ± 0.0^AB^	0.4 ± 0.0^B^
18:2n-6 *	3.0 ± 0.2^AB^	2.6 ± 0.1^A^	4.2 ± 0.3^BC^	3.5 ± 0.3^AB^	2.5 ± 0.1^A^	3.4 ± 0.4^ABC^	4.9 ± 0.4^BC^	2.2 ± 0.3^A^	12.4 ± 0.3^C^	3.4 ± 0.2^ABC^
18:3n-3	2.1 ± 0.2^A^	2.1 ± 0.1^A^	3.0 ± 0.1^BC^	2.1 ± 0.3^A^	2.4 ± 0.2^ABC^	2.6 ± 0.1^ABC^	3.4 ± 0.4^B^	2.2 ± 0.3^AC^	3.1 ± 0.2^BC^	0.6 ± 0.0^D^
18:4n-3 *	1.0 ± 0.1^ABC^	1.2 ± 0.1^ABC^	2.4 ± 0.2^A^	1.8 ± 0.4^AB^	1.7 ± 0.2^AB^	1.7 ± 0.3^AB^	3.0 ± 0.6^A^	0.6 ± 0.1^B^	0.8 ± 0.0^ABC^	0.5 ± 0.0^C^
∑20:1	1.6 ± 0.1^AD^	1.6 ± 0.1^A^	1.6 ± 0.1^AD^	0.8 ± 0.2^BEF^	0.5 ± 0.2^EF^	0.4 ± 0.0^EF^	0.4 ± 0.0^F^	1.5 ± 0.2^ABD^	3.3 ± 0.3^C^	1.1 ± 0.1^DE^
20:2NMI *	0.0 ± 0.0^AB^	0.0 ± 0.0^AB^	0.0 ± 0.0^A^	0.0 ± 0.0^A^	0.0 ± 0.0^A^	0.0 ± 0.0^ABC^	0.0 ± 0.0^AB^	0.0 ± 0.0^AB^	0.1 ± 0.0^BC^	0.8 ± 0.0^C^
20:2n-6	0.8 ± 0.0^A^	0.8 ± 0.0^A^	1.1 ± 0.1^B^	0.4 ± 0.0^C^	0.3 ± 0.1^CD^	0.3 ± 0.0^CD^	0.2 ± 0.0^CD^	0.3 ± 0.0^CD^	0.5 ± 0.0^C^	0.2 ± 0.0^D^
20:4n-6 *	2.2 ± 0.1^ACDE^	1.8 ± 0.1^ACD^	2.9 ± 0.1^ABE^	3.4 ± 0.2^BE^	3.7 ± 0.2^ABE^	7.6 ± 0.6^B^	8.5 ± 0.7^B^	2.8 ± 0.3^ABED^	0.6 ± 0.0^C^	1.2 ± 0.0^CD^
20:3n-3 *	1.1 ± 0.1^A^	1.2 ± 0.1^A^	1.1 ± 0.1^AB^	0.4 ± 0.0^ABCD^	0.3 ± 0.0^ABCDE^	0.2 ± 0.0^BCDE^	0.2 ± 0.0^CDE^	0.4 ± 0.0^ADE^	0.1 ± 0.0^DE^	0.0 ± 0.0^E^
20:4n-3	2.3 ± 0.2^AB^	2.3 ± 0.2^AB^	2.4 ± 0.1^AB^	2.9 ± 0.4^B^	2.3 ± 0.1^A^	1.8 ± 0.2^AB^	2.1 ± 0.2^AB^	1.3 ± 0.2^A^	0.4 ± 0.0^C^	0.4 ± 0.0^C^
20:5n-3	4.8 ± 0.5^AC^	4.1 ± 0.3^A^	8.3 ± 0.2^BD^	6.8 ± 0.4^DE^	9.2 ± 0.5^BF^	9.4 ± 0.2^BF^	8.0 ± 0.4^BDE^	6.1 ± 0.4^CE^	3.5 ± 0.2^A^	10.3 ± 0.3^F^
∑22:1 *	0.2 ± 0.0^AC^	0.2 ± 0.0^AC^	0.2 ± 0.0^ABC^	0.1 ± 0.0^AB^	0.1 ± 0.0^AB^	0.1 ± 0.0^AB^	0.0 ± 0.0^B^	0.3 ± 0.1^ABC^	2.6 ± 0.4^C^	0.4 ± 0.0^C^
22:4n-6	0.4 ± 0.0^AC^	0.4 ± 0.0^AC^	0.2 ± 0.0^AB^	0.2 ± 0.0^B^	0.2 ± 0.0^ABE^	0.6 ± 0.0^CD^	0.7 ± 0.1^D^	0.5 ± 0.1^CD^	0.1 ± 0.0^BE^	0.0 ± 0.0^E^
22:5n-6 *	1.2 ± 0.1^AB^	1.2 ± 0.0^A^	1.2 ± 0.1^ABD^	1.8 ± 0.2^AB^	3.2 ± 0.2^B^	2.4 ± 0.3^AB^	1.1 ± 0.1^ABCD^	1.5 ± 0.3^ABD^	0.2 ± 0.0^CD^	0.4 ± 0.0^D^
22:4n-3 *	0.8 ± 0.1^AC^	1.1 ± 0.1^A^	0.5 ± 0.1^ABC^	0.1 ± 0.0^BD^	0.1 ± 0.0^CD^	0.1 ± 0.0^CD^	0.1 ± 0.0^CD^	0.2 ± 0.0^AD^	0.0 ± 0.0^D^	0.0 ± 0.0^D^
22:5n-3	3.0 ± 0.2^A^	2.8 ± 0.1^A^	2.5 ± 0.2^AB^	1.9 ± 0.1^B^	1.8 ± 0.1^BC^	2.5 ± 0.1^AB^	2.8 ± 0.1^A^	2.9 ± 0.5^A^	1.0 ± 0.1^C^	2.6 ± 0.1^A^
22:6n-3 *	11.9 ± 0.9^A^	11.8 ± 0.2^A^	14.2 ± 0.9^AB^	22.2 ± 1.1^BCD^	31.1 ± 1.1^C^	25.5 ± 0.8^BCD^	14.5 ± 1.6^AD^	14.6 ± 1.7^AD^	8.7 ± 1.0^A^	25.5 ± 1.0^BCD^
24PUFA *	3.7 ± 0.5^AD^	3.0 ± 0.2^ACD^	5.2 ± 0.2^A^	0.5 ± 0.1^BE^	0.4 ± 0.1^BCE^	0.5 ± 0.1^BDE^	0.6 ± 0.1^BDE^	1.6 ± 0.3^BA^	0.2 ± 0.0^BE^	0.1 ± 0.0^E^

**Table 4:** Mean values of percentages (% of total fatty acids ± standard error) and total contents (∑FA, mg·g^−1^ dry weight) of fatty acids in food of studied fish: gut content of boganid charr *Salvelinus boganidae* (*S.b*. gut cont., number of samples, *n* = 2); Dryagin’s char *Salvelinus drjagini* (*S.d*. gut cont., *n* = 5); goggle-eyed charr (the form of *Salvelinus alpinus* complex) (g-e*S.a*. gut cont., *n* = 2); all from Lake Sobachye, diet of Arctic charr *Salvelinus alpinus* from the Ropsha farm (*S.a*. diet, *n* = 5); diet of brook trout *Salvelinus fontinalis* from the aquaculture of Timiryazev Academy (*S.f.* diet, *n* = 5). Normally distributed variables are compared by ANOVA and Tukey HSD post hoc test; the other variables marked with asterisk * and are compared by Kruskal–Wallis test. Means labelled with the same letter are not significantly different at *p* < 0.05 according to the relevant test. When Kruskal–Wallis test is insignificant, letter labels are absent.

	S.b. Gut Cont.			S.d. Gut Cont.			g-eS.a. Gut Cont.			S.a. Diet			S.f. Diet		
14:0	2.8	±	0.6^A^	5.3	±	0.5^B^	3.2	±	0.4^A^	2.3	±	0.0^A^	2.3	±	0.1^A^
15:0 *	0.3	±	0.0^AB^	0.4	±	0.0^A^	0.4	±	0.0^AB^	0.2	±	0.0^B^	0.2	±	0.0^B^
16:0	21.2	±	1.4^A^	15.3	±	0.6^B^	18.8	±	0.6^AD^	12.8	±	0.1^C^	17.0	±	0.2^BD^
16:1n-9 *	0.3	±	0.0^AB^	0.5	±	0.0^A^	0.5	±	0.1^AB^	0.1	±	0.0^AB^	0.1	±	0.0^B^
16:1n-7	4.5	±	0.9^AB^	6.0	±	1.1^A^	4.1	±	1.3^AB^	2.8	±	0.0^B^	2.2	±	0.1^B^
15-17BFA *	1.0	±	0.3^AB^	2.2	±	0.2^A^	1.2	±	0.0^AB^	0.2	±	0.0^B^	0.3	±	0.0^AB^
16PUFA	0.3	±	0.0^A^	0.9	±	0.2^AB^	0.3	±	0.1^A^	1.2	±	0.0^B^	0.7	±	0.0^AB^
17:0	0.3	±	0.0^AB^	0.3	±	0.0^B^	0.4	±	0.1^A^	0.2	±	0.0^C^	0.2	±	0.0^C^
∑17:1	0.2	±	0.0^A^	0.2	±	0.0^A^	0.2	±	0.1^A^	0.1	±	0.0^B^	0.1	±	0.0^B^
18:0	3.5	±	0.0^AB^	2.5	±	0.2^A^	5.6	±	1.8^B^	4.0	±	0.0^BC^	3.5	±	0.1^AC^
18:1n-9 *	9.1	±	0.2^A^	12.1	±	0.5^A^	8.2	±	0.3^A^	43.1	±	0.3^B^	40.5	±	0.4^AB^
18:1n-7	2.4	±	0.3^A^	2.4	±	0.1^A^	3.4	±	0.5^B^	3.1	±	0.0^BC^	2.7	±	0.1^AC^
18:2NMI *	0.0	±	0.0	0.0	±	0.0	0.0	±	0.0	0.0	±	0.0	0.0	±	0.0
18:2n-6	2.2	±	0.3^A^	4.4	±	0.4^B^	2.1	±	0.3^A^	14.6	±	0.1^C^	12.7	±	0.4^D^
18:3n-3	1.8	±	0.4^A^	3.1	±	0.2^B^	1.5	±	0.7^A^	5.6	±	0.1^C^	3.8	±	0.2^B^
18:4n-3 *	1.4	±	0.4^AB^	2.9	±	0.4^A^	1.6	±	1.0^AB^	0.6	±	0.0^AB^	0.5	±	0.0^B^
∑20:1 *	0.8	±	0.2^A^	1.2	±	0.1^AB^	0.9	±	0.2^A^	1.3	±	0.0^AB^	2.9	±	0.0^B^
20:2NMI *	0.0	±	0.0^A^	0.0	±	0.0^A^	0.0	±	0.0^A^	0.0	±	0.0^A^	0.1	±	0.0^A^
20:2n-6 *	0.7	±	0.2^AB^	1.0	±	0.1^A^	0.5	±	0.1^AB^	0.1	±	0.0^B^	0.1	±	0.0^AB^
20:4n-6 *	2.8	±	0.4^A^	2.0	±	0.1^A^	4.0	±	0.2^A^	0.3	±	0.0^AB^	0.2	±	0.0^B^
20:3n-3 *	1.0	±	0.5^AB^	1.3	±	0.1^A^	0.7	±	0.2^AB^	0.0	±	0.0^B^	0.0	±	0.0^B^
20:4n-3 *	1.5	±	0.4^AB^	2.1	±	0.2^A^	1.1	±	0.5^AB^	0.2	±	0.0^AB^	0.1	±	0.0^B^
20:5n-3	7.1	±	0.5^A^	7.9	±	0.3^A^	9.6	±	2.8^A^	3.0	±	0.1^B^	2.4	±	0.1^B^
∑22:1 *	0.1	±	0.1^AB^	0.2	±	0.0^A^	0.3	±	0.2^AB^	0.7	±	0.0^AB^	3.7	±	0.2^B^
22:4n-6 *	0.2	±	0.1^A^	0.2	±	0.0^A^	0.3	±	0.1^A^	0.0	±	0.0^A^	0.0	±	0.0^A^
22:5n-6	2.0	±	0.3^A^	1.3	±	0.1^B^	1.7	±	0.0^AB^	0.1	±	0.1^C^	0.0	±	0.0^C^
22:4n-3 *	0.9	±	0.5^AB^	1.6	±	0.2^A^	0.6	±	0.2^AB^	0.0	±	0.0^B^	0.0	±	0.0^B^
22:5n-3	2.1	±	0.2^A^	2.3	±	0.2^A^	2.7	±	0.1^A^	0.4	±	0.1^B^	0.3	±	0.0^B^
22:6n-3 *	23.6	±	5.9^A^	12.8	±	0.7^A^	20.4	±	1.1^A^	1.7	±	0.1^A^	1.8	±	0.1^A^
24PUFA *	2.0	±	1.4^AB^	3.3	±	0.4^A^	1.8	±	0.5^AB^	0.0	±	0.0^B^	0.0	±	0.0^B^
∑FA mg/g	24.6	±	5.1^A^	158.9	±	59.7^AB^	46.1	±	10.2^AB^	221.4	±	6.3^B^	111.4	±	6.6^AB^
